# Successful Hemostasis with Extended Half-life Recombinant Factor VIII in Circumcision

**DOI:** 10.4274/tjh.galenos.2019.2019.0305

**Published:** 2020-02-20

**Authors:** Başak Koç, Metin İshak Öztürk, Bülent Zülfikar

**Affiliations:** 1 İstanbul University Oncology Institute, Department of Pediatric Hematology-Oncology, İstanbul, Turkey; 2 Haydarpaşa Training and Research Hospital, Department of Urology, İstanbul, Turkey

**Keywords:** Hemophilia, Surgery, Circumcision, BAX 855

## To the Editor,

Intensified coagulation factor replacement is essential for surgical procedures in people with hemophilia A (HA). It is indicated during surgery and in the postoperative period [1,2]. The efficacy and the safety of PEGylated recombinant human full-length coagulation factor VIII (BAX 855) in prophylaxis and treatment of bleeding episodes have already been reported and its half-life in the circulation was proven to be 1.5 times longer compared to standard half-life FVIII (SHL-FVIII) [3]. Circumcision is a common surgical intervention in approximately half of the world [4,5]. In this report, we aimed to present our experience with extended half-life recombinant FVIII (EHL-rFVIII)-BAX 855 treatment for circumcision in two severe cases of HA.

The first patient was diagnosed at the age of 3 months with severe HA (factor VIII = 0.001 IU/mL=0.1%) with no family history. He started primary prophylaxis twice a week at the age of 17 months; however, his prophylaxis regimen had to be changed to 3 times a week at the age of 5 years old due to frequent bleeding of the elbows. He was enrolled in an EHL-rFVIII clinical trial at 5.5 years old, and the prophylaxis was continued twice a week for 3 years with no bleeding. The second patient was diagnosed at the age of 8 months with severe HA (factor VIII=0.003 IU/mL=0.3%); he had a family history. He began primary prophylaxis twice a week at the age of 15 months. He was enrolled in an EHL-rFVIII clinical trial at 5.5 years old, and prophylaxis was continued twice a week for 3 years with no bleeding. Both patients had no adverse events and no inhibitors during this period. The two patients were circumcised at 8 years old in a pediatric urology clinic. Both patients were hospitalized on the day of circumcision. One patient had phimosis and was hospitalized for 3 days; the other patient was hospitalized for 1 day. Both patients were circumcised under local anesthesia using a diathermic knife. Hemostasis control was achieved by tranexamic acid and EHL-rFVIII. Both patients were under prophylaxis at a dose of 45 IU/kg/day twice a week. The circumcisions were performed on the prophylaxis day, and 2 extra EHL-rFVIII doses (50 IU/kg/dose) were used during the prophylaxis regimen. Factor FVIII level was assessed by chromogenic assay on the first day of the circumcision. Factor VIII level was under 0.030 IU/mL for both patients at the beginning and 1.252 IU/mL (125.2%) and 2.180 IU/mL (218%) at 30 min, respectively. Both patients had regular wound healing. No unexpected bleeding or wound infections were recorded. They returned to their routine lives within 7 days.

Circumcision is a cultural and traditional surgical intervention, and many patients want to be circumcised around the world. In previously published series, it was reported that circumcision could be performed with minimal complication rates by using a diathermic knife. In this routine clinical practice, tranexamic acid and SHL-FVIII products have been used for hemostasis with decreasing doses between 4 and 14 days until wound healing occurs, depending on the severity of hemophilia [6,7]. In another protocol in which circumcision was performed under general anesthesia, fibrin glue application with 2-3 days of factor supplementation was found to be sufficient [8]. As we report here, just two extra doses of EHL-rFVIII were needed on postoperative days 1 and 2 for our patients who underwent circumcision. Our experiences with these two patients demonstrate that PEGylated rFVIII is well tolerated and efficacious for bleeding prophylaxis before circumcision.

There are limited data on EHL-rFVIII products in surgical interventions in the literature [9,10,11]. The first such prospective study reported 15 surgical interventions with PEGylated EHL-rFVIII and hemostatic efficacy was excellent for all subjects in both the intraoperative and perioperative period. Additionally, all interventions were scored as excellent postoperatively, except for one dental procedure that was graded as good. In addition, no related adverse events, including thrombosis and inhibitors, were recorded [9]. To the best of our knowledge, this is the first report to indicate two successful circumcision procedures performed under perioperative and postoperative EHL-rFVIII prophylaxis. Additionally, successful prophylaxis was achieved with lower frequency of factor supplementation compared to other series. As we mentioned above, no adverse events, no thrombotic events, and no inhibitor development were observed following prophylaxis.

In conclusion, EHL-rFVIII was safe and effective for circumcision management in cases of severe HA. Decreasing the amount and frequency of factor support seems to be possible according to this report.
